# Predicting intentions to seek help for depression among undergraduates in Sri Lanka

**DOI:** 10.1186/s12888-018-1700-4

**Published:** 2018-05-04

**Authors:** Santushi D. Amarasuriya, Anthony F. Jorm, Nicola J. Reavley

**Affiliations:** 1 Department of Medical Humanities, Faculty of Medicine, University of Colombo, PO Box 271, 25, Kynsey Road, Colombo 8, Sri Lanka; 2 Centre for Mental Health, Melbourne School of Population and Global Health, University of Melbourne, 207 Bouverie Street, Parkville, VIC 3010 Australia

**Keywords:** Help-seeking, Helping-seeking intentions, Mental health literacy, Depression, Professional help, Informal help, Undergraduate, University student, Sri Lanka

## Abstract

**Background:**

Studies have found that although there are high rates of depression among university students, their help-seeking practices are poor. It is important to identify students who are less likely to seek the necessary help, to encourage better help-seeking among them. This study, which was conducted among undergraduates in Sri Lanka, examined the associations between personal characteristics of the undergraduates and their intentions to seek help for depression.

**Methods:**

This was a cross-sectional study in which 4461 undergraduates (Male: *n* = 1358, 30.4%, Female: *n* = 3099, 69.5%; Mean age **=** 22.18; *SD* = 1.47) indicated their intentions to seek help if personally affected by depression, which was described in a hypothetical vignette about a peer experiencing depression symptomatology. The predictors of the undergraduates’ help-seeking intentions, including their sociodemographic characteristics, prior exposure to and recognition of the problem, and their stigma towards those with depression were examined using binary logistic regression analyses models.

**Results:**

The undergraduates’ ability to recognise the problem was one of the strongest predictors of their intentions to seek professional help. Those with higher levels of stigma were less likely to seek both professional and informal help. While females were less likely to consider professional help, they were more likely to consider the help of informal help-providers and to consider religious strategies. Medical undergraduates and those who had sought help for personal experiences of the problem were also more likely to consider informal help. However, all these associations resulted in small effect sizes, except for those between recognition of the problem and the undergraduates’ intentions to seek professional help, where medium to very large effect sizes were observed in the case of some the associations examined.

**Conclusions:**

Improvement of problem-recognition may be a key strategy for improving help-seeking among these undergraduates. Reduction of stigma may also be associated with better depression-related help-seeking of undergraduates. Females and medical undergraduates need to be educated about the importance of seeking appropriate types of help, and their informal social networks must be educated about how best to help them.

**Electronic supplementary material:**

The online version of this article (10.1186/s12888-018-1700-4) contains supplementary material, which is available to authorized users.

## Background

The high rates of depression found among university students [1, 2] highlight that many may require mental health support. While this indicates the need for examining the depression-related help-seeking practices of these students, it is also important to examine the predictors of such help-seeking and to identify those who are less likely to seek the necessary help.

In attempts to understand and predict help-seeking practices that university students might demonstrate if affected by a mental health problem, many studies have examined their help-seeking intentions, and likeliness and willingness to seek help for their problem [3–6]. The Theory of Planned Behaviour [7] provides strong support for the examination of help-seeking intentions as a predictor of actual help-seeking practices. In light of this, as part of a survey of depression-related literacy of undergraduates in Sri Lanka, Amarasuriya et al. [8] examined the intentions of these undergraduates to seek help if they were personally affected by depression. The condition was described using a vignette of an undergraduate who was exhibiting symptomatology of Major Depression as per the DSM-IV [9] and the participants were asked what they thought was wrong with the person in the vignette. Although only a low proportion of undergraduates were able to recognise the condition as being “depression”, as discussed in Amarasuriya et al. [9], this may have been due to the lack of lay terminology for the condition in the local languages [10, 11]. The following help-seeking actions were nominated by approximately ≥5% of the undergraduates as those they would engage in if they were affected by this problem (*N* = 4461): *Professional help*: psychiatrist/related help (7.3%), doctor/medical treatment (7.7%), counsellor/counselling (6.5%); *Informal help*: help from friends (34.2%), parents (20.2%), family (4.8%), persons not specified (13.9); *Self-help strategies*: doing enjoyable/relaxing/physical activities or taking a break (16.1%), dealing with educational difficulties/focussing on education/ managing work (9.0%); using religious-oriented strategies (7.2%) and understanding problem/self-initiated effort to get rid of problem (6.5%).

Although health pluralism is observed in Sri Lanka where, in addition to professional help, the population has endorsed a range of healers, such as alternative medicine practitioners [12, 13] and religious figures and practices [14, 15], the undergraduates in the Amarasuriya et al. study [9] mostly considered professional help and the help of their social network. However, it is noteworthy that when they were asked to rate the helpfulness of a list of potential help-providers, the clergy/religious priests were among those highly endorsed, indicating the presence of related treatment beliefs. However, these help-providers were not among those identified by the undergraduates in relation to their personal help-seeking intentions.

It is also noteworthy that although 9.3% of the undergraduates screened positive for Major Depression [8], which indicates that such students may require professional help, the findings as a whole show that the undergraduates are more likely to seek help from parents and friends than from professional sources, such as psychiatrists and counsellors [9]. This highlights the important roles the undergraduates’ informal social network may play in assisting them, including as a gate-way to treatment [16]. Their importance is further emphasized when considering the shortage of professional mental health services in the country [17]. It would be useful to identify any factors which predict the different help-seeking intentions of these undergraduates in order to understand those who should be targeted when attempting to promote appropriate help-seeking in this group.

Among factors found in previous studies to influence the help-seeking intentions of university students and young people are their gender [6, 18–21], stage of study and age [6, 18], ability to recognise their mental health problems [22], personal experiences of the problem or experiences of it through their family [18, 20, 23–25], and their stigmatising attitudes [26, 27]. However, findings regarding the associations between the help-seeking intentions of these young persons and the aforementioned factors have been sometimes inconsistent. For example, although many studies have found that female university students and adolescents show better help-seeking intentions [6, 18–21], the ability to recognise the problem has only sometimes been found to be associated with higher intentions to seek help among these groups [6, 22]. Furthermore, while certain stigmatising attitudes held by these groups have been associated with their lesser intentions to seek professional help, other types of stigma have been inversely associated with such help-seeking intentions [26, 27]. There is a dearth of published research examining how such factors may affect mental health-related help-seeking of Sri Lankan university students and the Sri Lankan population in general. One study found that the perceptions of carers of mentally ill patients regarding the stigma held by others was associated with delayed help-seeking of the patients they cared for [28]. More research is needed to understand the factors which may affect the help-seeking practices of university students in Sri Lanka. Accordingly, the aim of the present study was to examine the personal characteristics of undergraduates in Sri Lanka which predict their intentions to seek help for depression.

## Method

### Study design, setting and participants

The methodology of this cross-sectional study, which was conducted from June to November 2013, has already been described in Amarasuriya et al. [9, 29]. The study population consisted of undergraduates enrolled in a Sri Lankan university.

The study sites included five of the six undergraduate faculties of the University of Colombo, namely the Faculties of Arts and Education, Law, Management and Finance, Medicine, and Science, as well as the School of Computing, which is an affiliated institute of the university. The sampling strategy, which involved clustered sampling, aimed to produce as large a sample as possible by approaching all those attending a lecture (the cluster) which was common for each year of study in the research sites. In the case of the Faculty of Arts in which students had varied subject combinations, lectures with the largest student cohorts were approached for data collection. Data was not collected in the Faculty of Education, as the second and third year students of this faculty attended lectures in the Faculty of Arts, and as only fourth year students had lectures exclusively at this faculty. The strategy of systematically approaching students from all faculties and years of study during the identified lectures was considered to reduce any bias in sampling.

### Measures

Depression and depression literacy of the undergraduates was examined using a paper-based dual language questionnaire that was available in two versions, i.e., as English-Sinhala and English-Tamil versions. Participants could choose their preferred version. A vignette which described an undergraduate named ‘Z’, who exhibited symptoms of Major Depression as per the Diagnostic and Statistical Manual of Mental Disorders-IV, was provided as the problem trigger. The present paper examines the predictors of the undergraduates’ personal help-seeking intentions. Therefore, it only describes the questionnaire items relevant to this aim. The complete depression literacy questionnaire, including the depression vignette that was used and details regarding its development, have been previously published [9, 29]. The questionnaire has been provided as Additional file 1 for the convenience of the reader.

#### Help-seeking intentions

The undergraduates’ help-seeking intentions were elicited in relation to the depression vignette using the following open-ended question: “If you have this problem, what would you do?” Multiple responses were possible.

#### Problem recognition

This was examined using the following open-ended question: “What do you think is wrong with ‘Z’?” Multiple responses were possible. Amarasuriya et al. [29] categorised these responses as follows: recognised as depression, recognised using other mental health-related labels (pertaining to the label categories “mental illness”, “stress/pressure/mental suffering”, “mental issue”), and not recognized (where responses were not relevant to either of the two previous response categories).

#### Screening positive for major depression

The Patient Health Questionnaire-9 (PHQ-9) is a self-administered measure which consists of nine items, based on the nine symptoms of Major Depression in the DSM-IV (Kroenke & Spitzer, 2002). This measure instructed respondents to rate the degree to which they were bothered by the symptoms presented in the measure during the previous two weeks, using a four point scale consisting of the following rating options: *Not at all*, *Several days*, *More than half the days*, *Nearly every day*. These rating options were scored from 0 to 3 respectively. Therefore, a maximum score of 27 could be obtained. If five of the nine symptoms were found to be present for *More than half the days* or *Nearly every day*, then a diagnosis of *Major Depression* was given. However, for these diagnoses to be given, either the symptom relating to depressed mood or anhedonia had to be present. If the symptom relating to suicidal thoughts was present at all, it was then considered within the symptom count for these diagnoses.

#### Stigmatizing attitudes

The questionnaire examined the undergraduates’ stigmatising attitudes towards those with depression, by examining their negative attitudes towards ‘Z’ using the Personal Stigma Scale, and their willingness to interact with ‘Z’ using the Social Distance Scale.

The Personal Stigma Scale is a component of the Depression Stigma Scale [30]. This study used a version of this scale adapted for use among young persons between 12 and 25 years of age (Jorm & Wright, 2008). The scale consisted of seven statements and asked participants to indicate how much they agreed or disagreed with the statements, by using a five-point rating scale (scored from 1 to 5) consisting of the following rating options: *Strongly agree*, *Agree*, *Neither agree nor disagree*, *Disagree*, *Strongly disagree*. The items were reverse-scored so that higher scores indicated a higher level of personal stigma. A total score of 35 could be obtained.

The Social Distance Scale which was used is based on a social distance scale for adults [31], that was later adapted for use among a population of young persons between 12 and 25 years of age (Jorm & Wright, 2008). The scale consists of five items which ask participants to indicate their willingness to do the activities which are listed with ‘Z’, using a four-point rating scale (scored from 1 to 4), consisting of the following rating options: *Yes, definitely*, *Yes, probably*, *Probably not*, *Definitely not*. Higher scores on the scale indicate lesser willingness to interact with ‘Z’ and hence, greater desire for social distance from ‘Z’. A total score of 20 could be obtained.

Amarasuriya et al. [32] found that the Personal Stigma scale consisted of two dimensions of stigma, i.e., the Weak-not-Sick and Dangerous-Undesirable dimensions (consisting of three items each), while the Social Distance scale consisted of one dimension (consisting of five items), which was labelled as Social Distance. This led to the construction of three subscales which reflected these three dimensions of stigma (see Amarasuriya et al. [32] for details regarding construction of the scales, factor loadings of scale items, reliabilities of scales and their limitations relating to low reliability estimates).

#### Exposure to problem and help-seeking

Three closed questions were used to examine the undergraduates’ previous experiences of the problem and their related help-seeking practices. Participants were asked if anyone in their family or close circle of friends had experienced a problem like ‘Z’s (response options: *Yes*, *No*, *Don’t know*), if they had ever had a problem like ‘Z’s (response options: *Yes*, *No*, *Don’t know*), and in the case of responding as ‘*Yes’* or ‘*don’t know’* to the latter, if they had dealt with the problem on their own, without getting help from others (response options: *Yes*, *Tried first but got help later, No*).

#### Demographic characteristics

The following variables were examined: faculty of study (open-ended question), gender, year of study, age, religion (response options: *Buddhist, Hindu, Islamic, Christian, Roman Catholic, Atheist, Other*) and residence (response options: *at home, hostel, rented place, home of friend/relative, other*).

### Procedure

The questionnaires were distributed to the undergraduates during lectures. Participation was voluntary and anonymous. Implied consent procedures were used, where consent to participate was implied when a filled questionnaire was returned. The undergraduates took approximately 20 min to complete the questionnaire.

### Statistical analysis

The aggregates for all types of professional and informal help that the undergraduates identified as those they would seek if personally affected by the problem were calculated. The category “person not specified” was excluded from the aggregate for informal help due to the ambiguity of this response. The aggregate for help-seeking intentions relating to self-help strategies was not calculated, as there were a range of differing responses, some of which may have been unhelpful when dealing with depression. The correlation between the aggregates for intentions to seek professional and informal help was also found using the phi correlation coefficient.

Binary logistic regression models were used to examine the predictors (IVs) of each of the aforementioned help-seeking intentions of the undergraduates (DVs) (i.e., professional help, informal help and self-help strategies). The following categorical variables were simultaneously entered into binary logistic regression models to examine the predictors of each of these types of help-providers/strategies of help-seeking, where the variable sub-categories which are italicised were the reference categories for each of the respective variables: gender (*male*, female); faculty of study (*Medicine*, Arts and Education, Law, Management and Finance, Science, School of Computing); year of study (*1st year*, 2nd year, 3rd year, 4th year, 5th year Medicine); age category (*18–20 years*, 21–23 years, 24 years and above); ability to recognise the problem in vignette (*not recognised*, recognised as depression, recognised using other mental health-related labels (pertaining to the label categories “mental illness”, “stress/pressure/mental suffering”, “mental issue”); if the respondents screened positive for Major Depression as per the PHQ-9 (*no*, yes); if the problem in the vignette had been experienced by family or friends (*no*, yes, don’t know); if the problem was personally experienced (*no*, yes, don’t know); and from those who responded as *yes* or *don’t know* to the latter question, if they had sought help for the problem (*help not sought*, tried first but got help later, help sought; those who did not indicate personal experience of the problem were included in the analysis, but were dummy coded as a *not relevant* category). The stigma scale scores, which were continuous variables, were entered into the model in relation to the Weak-not-Sick, Dangerous-Undesirable and Social Distance scales that have been described in Amarasuriya et al. [32]. As simultaneous regression analysis models were used, this allowed for an examination of each of these variables as predictors of the undergraduates’ help-seeking intentions while adjusting for all the other variables entered into the model. The analyses were also adjusted for the participants’ religion, residence and language of response. Given the large number of predictors entered into each of the regression models, the *p* < .01 significance level was used to reduce the Type I error rate.

In order to examine the effect sizes of these associations, in instances in which the ORs were < 1, these were converted to 1/OR to place all effects in a common frame of reference [33]. ORs of 1.5 were considered as small effect sizes, 2.5 as medium effect sizes, 4 as a large effect sizes and 10 as very large effect sizes [34]. The results were interpreted in relation to these effect size estimates. As described in Mackinnon [35], the dichotomization of continuous scaled predictors for use in logistic regression analyses reduces the power of these scaled predictors. Therefore, as recommended by Mackinnon, in the case of the stigma scores which were continuous scaled predictors, Interquartile Odds Ratios (IQOR) which retain the full power of these predictors were calculated. This placed these variables within a common frame of reference with the other predictor variables when examining their effect sizes.

It must be noted that problem recognition as a predictor of intentions to seek help from professional help-providers has already been examined in Amarasuriya et al. [9]. The present analysis adjusted for a larger number of variables (stigma, exposure to problem and whether help was sought if problem was personally experienced), and also examined whether problem-recognition is a predictor of the undergraduates’ intentions to seek help from informal help-providers or to engage in the self-help strategies identified.

Data was analysed using the Statistical Package for the Social Sciences, version 23.

## Results

From a total of 4671 undergraduates who participated in the depression literacy survey, 4461 indicated their help-seeking intentions if personally affected by the problem described in the case vignette. Sample characteristics of those who indicated their help-seeking intentions are provided in Table 1. Interquartile ranges (IQR) for the stigma scale scores were as follows: Weak-not-Sick: IQR = 3, Dangerous-Undesirable: IQR = 2 and Social Distance: IQR = 4.

**Table 1:** Demographic and other characteristics of undergraduates who indicated their personal help-seeking intentions (*n* = 4461)

Variables	n	%
Gender		
Male	1358	30.4
Female	3099	69.5
Faculty		
Medicine	581	13.0
Arts and Education	1146	25.7
Law	601	13.5
Management and Finance	993	22.3
Science	638	14.3
School of Computing	501	11.2
Year of Study		
1st year	1845	41.4
2nd year	1188	26.6
3rd year	802	18.0
4th year	516	11.6
5th year (Medicine)^a^	110	2.5
Age group (Mean = 22.18; *SD* = 1.47)		
18–20 years	486	10.9
21–23 years	3199	71.7
24 and above	771	17.3
Ethnicity		
Sinhala	4096	91.8
Tamil	176	3.9
Sri Lankan Moor	140	3.1
Other	45	1.0
Religion		
Buddhism	3889	87.2
Hinduism	145	3.3
Islam	144	3.2
Roman Catholicism	206	4.6
Other	71	1.6
Residence when going to University		
Home	1684	37.7
Hostel	1340	30.0
Rented place	1125	25.2
Home of friend or relative	258	5.8
Other	50	1.1
Screening positive for Major Depression^b^ (*n* = 4123)		
No	3747	90.9
Yes	376	9.1
Exposure to problem through family/ friends		
No	1700	38.1
Yes	1651	37.0
Don’t know	982	22.0
Personal experience of problem		
No	2409	54.0
Yes	1463	32.8
Don’t know	308	6.9
If problem personally experienced (responding as ‘Yes’ or ‘Don’t know), if help sought (*n* = 1771)		
Help not sought	658	37.2
Tried first but got help later	683	38.6
Help sought	364	20.6

Notes

^a^Only the Faculty of Medicine has a 5th year of study

^b^Only responses which had ≤ 1 missing response on the PHQ-9 were considered valid for this variable

Percentages do not total to 100% due to missing values

Overall, 23.5% undergraduates indicated that they would use some type of professional option of help, while 45.5% indicated intentions to use some type of informal help. A weak negative correlation was found between the aggregates of the undergraduates’ intentions to seek professional and informal help (phi correlation coefficient = − 0.10, *p* < .001).

Subsequent to exclusion of cases due to missing values of the studied variables, the predictors of the help-seeking intentions of 3760 undergraduates were examined. The adjusted odds ratios (ORs) for the variables which were found to be significant predictors of the undergraduates’ intentions to seek professional/formal help, to seek informal help and to engage in self-help/other strategies are presented in Tables 2, 3 and 4 respectively. The IQORs for instances in which significant associations were found between the stigma scale scores and the undergraduates’ help-seeking intentions are also provided in these tables. The Nagelkerke’s R^2^, which is a generalization of the coefficient of determination in linear regression to logistic models and useful for comparing models within the same dataset is reported in relation to each of the analyses [36].

**Table 2:** Predictors of intending to seek professional help examined using simultaneous binary logistic regression (n = 3760)

Predictor variable	Psychiatrist/related help	Doctor/medical treatment	Counsellor/counselling	All types of professional help
	Adjusted OR (99% CI)	Adjusted OR (99% CI)	Adjusted OR (99% CI)	Adjusted OR (99% CI)
Gender (reference group: Male)				
Female	0.65^**^ (0.44, 0.96) [Equiv OR = 1.54]			0.72^**^ (0.57, 0.92) [Equiv OR = 1.39]
Faculty (reference group: Medicine)				
Arts and Education				1.87^**^ (1.16, 3.00)
Exposure to problem through family and friends (reference group: response: no)				
Response: Yes		0.64^**^ (0.42, 0.96) [Equiv OR = 1.56]		
Recognition of problem (reference group: not recognised)				
Recognised as depression	12.85^***^ (6.03, 27.41)	1.90^**^ (1.07, 3.40)	2.34^***^ (1.29, 4.23)	3.53^***^ (2.44, 5.12)
Recognised using mental health-related labels (excluding the label “depression”)	4.16^***^ (2.19, 7.92)	1.87^***^ (1.21, 2.90)	1.70^**^ (1.06, 2.72)	2.30^***^ (1.74, 3.06)
Stigma Scale Scores				
Weak-not-Sick Scale	0.86^***^ (0.80, 0.92) [IQOR = 0.63] [Equiv OR = 1.59]	0.86^***^ (0.80, 0.92) [IQOR = 0.63] [Equiv OR = 1.59]	0.92^**^ (0.86, 1.00) [IQOR = 0.79] [Equiv OR = 1.26]	0.86^***^ (0.82, 0.90) [IQOR = 0.64] [Equiv OR = 1.56]
Nagelkerke R^2^	0.16	0.08	0.05	0.12

**p < .01; ***p < .001

Notes

Interquartile ORs abbreviated as IQOR

ORs < 1 were converted into 1/OR and have been presented as Equiv OR

Only adjusted ORs for the variables which were found to be significant predictors of the undergraduates’ intentions to seek professional help have been provided

**Table 3:** Predictors of intending to seek help from informal help-providers examined using simultaneous binary logistic regression (*n* = 3760)

Predictor variable	Friend	Parent	Family	All types of informal help
	Adjusted OR (99% CI)	Adjusted OR (99% CI)	Adjusted OR (99% CI)	Adjusted OR (99% CI)
Gender (reference group: Male)				
Female		2.22^***^ (1.68, 2.94)		1.51^***^ (1.23, 1.86)
Faculty (reference group: Medicine)				
Arts and Education	0.61^**^ (0.41, 0.92) [Equiv OR = 1.64]			0.60^**^ (0.41, 0.90) [Equiv OR = 1.67]
Management and Finance	0.65^**^ (0.45, 0.96) [Equiv OR = 1.54]	0.59^**^ (0.38, 0.93) [Equiv OR = 1.69]	0.46^**^ (0.22, 0.97) [Equiv OR = 2.17]	0.61^**^ (0.42, 0.89) [Equiv OR = 1.64]
Computer	0.62^**^ (0.40, 0.94) [Equiv OR = 1.61]			0.66^**^ (0.44, 0.98) [Equiv OR = 1.52]
Year of study (reference group: 1st year)				
3rd Year		0.70^**^ (0.49, 0.98) [Equiv OR = 1.43]		
Whether those who personally experienced problem sought help (reference group: help not sought)				
Tried first but got help later	1.74^***^ (1.25, 2.43)	1.95^***^ (1.25, 3.05)		1.83^***^ (1.33, 2.52)
Help sought	1.91^***^ (1.28, 2.84)	1.76^***^ (1.05, 2.98)		1.94^***^ (1.32, 2.86)
Recognition of problem (reference group: not recognised)				
Recognised using mental health-related labels (excluding the label “depression”)		1.33^**^ (1.02, 1.74)		
Stigma Scale Scores				
Dangerous-Undesirable Scale	0.95^**^ (0.90, 1.00) [IQOR = 0.90] [Equiv OR = 1.11]	0.93^**^ (0.87, 0.99) [IQOR = 0.86] [Equiv OR = 1.16]		0.94^**^ (0.89, 0.98) [IQOR = 0.87] [Equiv OR = 1.15]
Social Distance Scale	0.94^***^ (0.90, 0.97) [IQOR = 0.77] [Equiv OR = 1.30]			0.96^**^ (0.93,1.00) [IQOR = 0.86] [Equiv OR = 1.16]
Nagelkerke R^2^	0.04	0.10	0.05	0.06

^**^ *p < .01;* ^***^ *p < .001*

Notes

Interquartile ORs abbreviated as IQOR

ORs < 1 were converted into 1/OR and have been presented as Equiv OR

Only adjusted ORs for the variables which were found to be significant predictors of the undergraduates’ intentions to seek informal help have been provided

**Table 4:** Predictors of intending to engage in self-help/other strategies examined using simultaneous binary logistic regression (n = 3760)

Predictor variable	Enjoyable/relaxing/physical activities or taking break	Using religious-oriented strategies	Dealing with educational difficulties	Understand problem/self-initiated effort to resolve it
	Adjusted OR (99% CI)	Adjusted OR (99% CI)	Adjusted OR (99% CI)	Adjusted OR (99% CI)
Gender (reference group: Male)				
Female		1.73^**^ (1.11, 2.70)	1.72^***^ (1.17, 2.52)	
Faculty (reference group: Medicine)				
Arts and Education			0.39^**^ (0.19, 0.79) [Equiv OR = 2.56]	
Year of study (reference group: 1st year)				
5th Year (Medicine)	0.22^**^ (0.05, 0.96) [Equiv OR = 4.54]			
Whether those who personally experienced the problem sought help (reference group: help not sought)				
Help sought	0.55^**^ (0.33, 0.90) [Equiv OR = 1.82]			0.26^**^ (0.09, 0.76) [Equiv OR = 3.85]
Screening positive for Major Depression (reference group: No)				
Yes			0.55^**^ (0.30, 1.00) [Equiv OR = 1.82]	
Recognition of problem (reference group: not recognised)				
Recognised as depression	1.78^***^ (1.20, 2.63)		0.20^***^ (0.10, 0.39) [Equiv OR = 5.00]	
Recognised using mental health-related labels (excluding the label “depression”)	1.44^**^ (1.07, 1.92)		0.47^***^ (0.34, 0.66) [Equiv OR = 2.13]	
Stigma Scale Scores				
Weak-not-Sick Scale			1.10^**^ (1.02, 1.18) [IQOR = 1.33]	
Nagelkerke R^2^	0.06	0.06	0.09	0.04

^**^ *p < .01;* ^***^ *p < .001*

Notes

Interquartile ORs abbreviated as IQOR

ORs < 1 were converted into 1/OR and have been presented as Equiv OR

Only adjusted ORs for the variables which were found to be significant predictors of the undergraduates’ intentions to seek self-help strategies have been provided

Following is a description of the significant effects found, including an interpretation of the effect sizes of the ORs/IQORs that were obtained.

### Predictors of intending to seek professional/formal help

Those who recognized the problem as depression had higher odds of seeking psychiatric help, (OR = 12.85), while such problem recognition was also associated with higher odds of considering all types of professional help (OR = 3.53). These associations resulted in very large and medium effect sizes respectively. Recognition of the problem using a mental health-related label (excluding “depression”) was associated with higher odds of considering the help of psychiatrists (OR = 4.16) and resulted in a large effect size.

All other significant associations found between the undergraduates’ intentions to seek professional/formal help and the examined factors had small effect sizes. Relatedly, while those who recognised the problem as depression, or by using a mental health-related label had higher odds of considering the help of doctors and counsellors, those who used mental health-related labels had higher odds of considering professional help in general; those in the Faculty of Arts and Education (compared to medical undergraduates) had higher odds of intending to seek professional help in general; female undergraduates had lower odds of considering the help of psychiatrists and professional help in general; those who had been exposed to the problem through family and friends had lower odds of considering the help of doctors/to get medical assistance; and those with higher scores on the Weak-not-Sick scale had lower odds of intending to seek all types of professional help.

### Predictors of intending to seek informal help

All significant associations which were observed resulted in small effect sizes. Relatedly, female undergraduates had higher odds of considering the help of their parents and informal help in general; those who had sought help for the problem or had sought help after trying to deal with it alone had higher odds of considering the help of both the aforementioned help-providers as well as their friends; those using mental health-related labels for the problem (excluding the label “depression”) had higher odds of indicating intentions to seek help from their parents, while those in the third year had lower odds of intending to seek such help; those in the Faculties of Arts and Education, Management and Finance and the School of Computing (compared to Medical undergraduates) had lower odds of intending to seek help from their friends and of seeking informal help in general; Management and Finance undergraduates also had lower odds of intending to seek help from their parents and family; those with higher scores on the Dangerous-Undesirable and Social Distance scales had lower odds of considering informal help in general as well as the help of their friends, while those with higher scores on the Dangerous-Undesirable scale also had lower odds of indicating intentions to seek help from their parents.

### Predictors of intending to engage in self-help/other strategies

A few of the examined associations resulted in medium to large effect sizes. Those who recognised the problem as “depression” had lower odds of indicating that they would deal with their educational difficulties (OR = 0.20; Equivalent OR for estimating effect size of association = 5.0), while 5th year Medical undergraduates had lower odds of indicating their intentions to engage in enjoyable/relaxing/physical activities or of taking a break (OR = 0.22; Equivalent OR for estimating effect size of association = 4.54); Both these associations resulted in large effect sizes. Those who sought help for their problem had lower odds of intending to understand the problem/of engaging in self-initiated efforts to resolve the problem (OR = 0.26; Equivalent OR for estimating effect size of association = 3.85), while those from the Faculties of Arts and Education (compared to Medicine) had lower odds of indicating that they would deal with their educational difficulties (OR = 0.39; Equivalent OR for estimating effect size of association = 2.56); These latter two associations resulted in medium effect sizes.

All other significant associations which were observed resulted in small effect sizes. Relatedly, females had higher odds of intending to use religious-oriented strategies. Females and those with higher scores on the Weak-not-Sick Scale had higher odds of indicating that they would deal with their educational difficulties, while the odds of using this strategy were lower for those who screened positive for Major Depression and those who recognised the problem by using other mental health-related labels. While the odds of intending to engage in enjoyable/relaxing/physical activities or of taking a break was higher among those who recognised the problem as “depression” or used other mental health-related labels for this purpose, odds of considering such help was lower among those who sought help when they personally experienced the problem.

## Discussion

This study attempts to identify some of the personal characteristics of the undergraduates which predict their help-seeking intentions. The findings indicate that while those who recognise the problem have greater intentions to seek professional assistance, those with various stigmatising attitudes show lesser intentions to seek both professional and informal help. The findings also indicate that those who have sought help for their personal experiences of the problem are more likely to consider the help of informal help-providers. There is some evidence that female undergraduates are less likely to seek professional assistance and more likely to seek help from informal help-providers and to engage in informal strategies of help. Medical undergraduates are also more likely to seek help from their informal social network. Some of the examined variables also predicted the undergraduates’ intentions to engage in some of the self-help strategies, where these associations ranged from medium to large effect sizes. However, it must be noted that a majority of the examined associations resulted in small effect sizes.

While those who recognised the problem as “depression” were more likely to consider psychiatric help (very large effect size) as well as professional help in general (medium effect size), recognition using mental health-related labels was also associated with being more likely to seek psychiatric help (large effect size). The results highlight that problem recognition is a key predictor of the undergraduates’ intentions to seek professional help. Recognition of the problem as “depression” was the strongest predictor of the undergraduates’ intentions to seek any type of help. This study supports previous findings which have indicated that accurate use of psychiatric labels is associated with preference to seek professional help among young people [22]. While the present findings indicate that the undergraduates’ ability to recognise depression is important to their help-seeking, they highlight that the low rates of depression recognition found in this population need attention [9]. However, as discussed in Amarasuriya et al., [9], the low rates of depression recognition found may be due to the lack of lay terminology for the condition in Sri Lanka. This highlights the challenges that may be faced in initiatives to improve the use of the depression label in this population. Therefore, as an initial step, the undergraduates should be helped to at least recognise the problem as being mental health-related, as this may also be related to improved help-seeking among them. Although depression recognition was associated with poorer intentions to deal with educational difficulties and resulted in a large effect size, the results also indicate that recognition was associated with higher intentions to engage in enjoyable/relaxing activities (small effect size), which have been found to be effective self-help strategies for dealing with depression [37].

Many of the other significant associations that were observed between the examined variables and the help-seeking intentions of the undergraduates resulted in small effect sizes, except for a few concerning the undergraduates’ intentions to engage in self-help strategies that resulted in medium to large effect sizes. However, it would be useful to consider how all these associations may influence the help-seeking practices of this population. The findings indicate that stigmatising attitudes of the undergraduates towards those who are depressed may be negatively associated with their personal help-seeking. While stigma that the problem is a “weakness” and not a “sickness” seems to be associated with lesser acknowledgement of the need for professional help, other stigmatising attitudes, such as the perception that those with depression are “dangerous” and “undesirable” and the desire for social distance from such individuals, may also be negatively associated with these undergraduates’ attempts to seek help from their informal help-providers. The findings indicate that the examined dimensions of stigma could have a consistently negative effect on the help-seeking of these undergraduates. The current findings together with recent findings from Sri Lanka, that perceived stigma of carers of mentally ill patients may result in help-seeking delays of these patients [28], highlight how the different dimensions of mental health-related stigma might have detrimental effects on the help-seeking practices of the Sri Lankan population.

The findings indicate that females may be less likely than males to seek professional help if personally affected by the problem. This finding does not align with the findings of many previous studies among university students in other countries, which have found instead that males are more reluctant to seek professional help for their problems [6, 18–21], where this may be associated with their identification with traditional masculinity roles [38–40]. However, in Asian cultures in which there may be greater reliance on family and friends when dealing with mental health problems, females may be more influenced by such cultural influences in their help-seeking practices [41, 42]. The findings also provide evidence of the female undergraduates’ greater reliance on social support, such as from their parents, and their greater endorsement of informal strategies of help, such as religious-oriented activities, when dealing with the problem. These findings may be highlighting a greater tendency for female undergraduates to first activate such socially-sanctioned informal pathways of help when attempting to deal with their mental health problems. Alternatively, such greater endorsement of informal help by females may be reflecting their greater preference for emotional support and person-centred and social-network-related help when attempting to deal with the problem [43–45].

While the medical undergraduates seem to be more likely to consider informal help for the problem than their non-medical counterparts, they do not show any difference in their inclination to seek professional help, despite their medical training. In fact, intentions to seek professional help were lower among the medical students as compared to those in the Arts and Education Faculties. Many previous studies have shown that professional help-seeking among medical students is low [46–48] and that they prefer to seek help from informal sources [47, 49–51]. A further analysis of the help-seeking intentions of medical vs. non-medical undergraduates is presented in Amarasuriya et al. [52]. Other noteworthy findings were that those in the Medical Faculty were more likely to focus on dealing with education-related difficulties than those in the Arts Faculty, and that those in the 5th year of Medicine were less likely to consider engaging in relaxing and enjoyable activities and that these associations resulted in medium to large effect sizes. Both these findings may be reflective of the academic and time-related pressures on those undertaking medical training.

Data was not obtained regarding the types of help sought by those who reported previous experiences of the problem and related help-seeking. However, the results indicate that previous experiences of seeking help for the problem are associated with being less likely to try to understand the problem and/or engage in self-initiated efforts to deal with the problem (medium effect size). Therefore, those who have sought help for previous personal experiences of the problem seem to be less inclined to personally deal with the problem. There were also no significant associations found between such previous help-seeking and the undergraduates’ intentions to seek professional help. However, the findings did indicate that those who had sought help previously were more likely to indicate that they would seek informal help for the problem, such as from parents and friends. Such findings could be indicating that these undergraduates’ past help-seeking experiences with these informal help-providers were positive. This would be encouraging, given the inclination of the undergraduates to seek such informal help for their problems [9]. However, such an association is only speculative at present.

Other findings relating to students who indicated that they had experienced or were experiencing the problem were nevertheless discouraging. For example, those who screened positive for Major Depression did not differ from those screening negative with regard to their intentions to seek help from both the formal and informal help-providers. Furthermore, while those who had personally experienced the problem were less likely to consider self-help strategies, such as engagement in relaxing and enjoyable activities, those who were exposed to the problem through their family or friends were less likely to consider the help of doctors. These findings concur with the findings of many previous studies which have found that the personal experience of depression or other mental health problems, or experiences of these through family members, could be negatively associated with help-seeking among university students [20, 23–25]. Such findings are concerning, as they indicate that those who are most likely to be in need of help may not seek it. Therefore, it is important to address factors which may act as barriers and impede the help-seeking of these groups and improve contact between suitable help-providers and the affected groups. As seen in many previous studies, such experiences of seeking help may in turn improve the subsequent help-seeking practices of these university students [21, 41].

The undergraduates could benefit from the support of both professional help-providers, in receiving the necessary treatment and interventions for their depression, and their informal social networks, with regard to provision of care, emotional support and encouragement/support to seek professional help. However, overall, the help-seeking pattern of the undergraduates indicates that those who seek professional help have a lesser tendency to seek help from their informal help-providers, while those who seek help from their informal help-providers have a lesser tendency to seek help from professional help-providers. Therefore, it may be necessary to educate these undergraduates about the importance of reaching out to these different sources of help and support. Furthermore, given the evidence that those who seek help from informal help-providers are less inclined to seek professional help, it is also important to educate the social networks of these undergraduates about the need for professional help for depression, as they may play pivotal roles as gate-way providers to treatment [16].

The findings need to be considered in light of the limitations of the study. The depression vignette is limited in its ability to capture the different factors which could influence the help-seeking intentions of the undergraduates in real-life. Furthermore, the methodology of open-ended questions was dependent on the undergraduates’ motivation to provide descriptive and comprehensive responses relating to their help-seeking intentions. However, such methodology could be considered to reflect the cognitive mechanisms that the undergraduates would undergo in deciding how to deal with the problem, and therefore to be a suitable strategy to examine their help-seeking intentions. It is also necessary to note that many of the predictors of the undergraduates’ help-seeking intentions were associated with small effect sizes [33]. Furthermore, the predictors which were examined only accounted for a small percentage of variance in the undergraduates’ help-seeking intentions, indicating that there were other factors influencing these variables that had not been considered in the study. The use of qualitative methodology in the future, such as in-depth interviews, would help in gaining a more comprehensive understanding of the help-seeking practices of the undergraduates and the factors which influence these practices. Although data was collected only from one University in Sri Lanka, the large sample size, including undergraduates from diverse disciplines and all years of study, indicate that the findings are useful in identifying the personal characteristics which predict intentions to seek help for depression among undergraduates in Sri Lanka.

## Conclusions

The findings indicate that among the personal characteristics of the undergraduates that were examined, their ability to recognize the problem is a key predictor of their inclination to seek help. Reduction of their stigma may also be associated with such an outcome. There is also some evidence indicating that both female and medical undergraduates are inclined to seek informal help and that their intentions to seek professional help need to be improved. Such findings emphasise the importance of educating these groups about the need to seek help for their depression, as well as educating the informal help-providers that they approach about how best to assist these undergraduates.

## Additional file

Additional file 1: English-Sinhala version of questionnaire (PDF 189 kb)

## Abbreviations

IQOR: Interquartile odds ratio
IQR: Interquartile range
